# Bypassing health facilities for childbirth: a multilevel study in three districts of Gujarat, India

**DOI:** 10.3402/gha.v9.32178

**Published:** 2016-08-19

**Authors:** Mariano Salazar, Kranti Vora, Ayesha De Costa

**Affiliations:** 1 Department of Public Health Sciences, Karolinska Institutet, Stockholm, Sweden; 2 Department of Reproductive and Child Health, Indian Institute of Public Health, Ahmedabad, India

**Keywords:** India, bypassing, multilevel, EmOC, childbirth

## Abstract

**Background:**

Bypassing available facilities for childbirth has important implications for maternal health service delivery and human resources within a health system. The results are the additional expenses imposed on the woman and her family, as well as the inefficient use of health system resources. Bypassing often indicates a lack of confidence in the care provided by the facility nearest to the mother, which implies a level of dysfunctionality that the health system needs to address. Over the past decade, India has experienced a steep rise in the proportion of facility births. The initiation of programs promoting facility births resulted in a rise from 39% in 2005 to 85% in 2014. There have been no reports on bypassing facilities for childbirth from India. In the context of steeply rising facility births, it is important to quantify the occurrence of and study the relative contributions of maternal characteristics and facility functionality to bypassing.

**Objectives:**

1) To determine the extent of bypassing health facilities for childbirth among rural mothers in three districts of Gujarat, India, 2) to identify associations between the functionality of an obstetric care (OC) facility and it being bypassed, and 3) to assess the relative contribution of maternal and facility characteristics to bypassing.

**Design:**

A cross-sectional survey of 166 public and private OC facilities reporting ≥30 births in the 3 months before the survey was conducted in three purposively selected districts (Dahod, Sabarkantha, and Surendranagar) in the state of Gujarat, India. Besides information on each facility, data from 946 women giving birth at these facilities were also gathered. Data were analyzed using a multilevel mixed-effects logistic regression model.

**Results:**

Off all mothers, 37.7% bypassed their nearest facility for childbirth. After adjusting for maternal characteristics, for every one-unit increase in the facility's emergency obstetric care (EmOC) signal functions, the odds of bypassing a facility for childbirth decreased by 37% (adjusted odds ratio [AOR] 0.63, 95% confidence interval [CI]: 0.53–0.76).

**Conclusions:**

This study shows that independent of maternal characteristics, in our setting, women will bypass obstetric facilities that are not adequately functional, and travel further to others that are more functional. It is important that the health system should focus on facility functionality, especially in the context of sharply rising hospital births.

## Introduction

Bypassing health facilities for childbirth is said to occur if women choose to give birth in a facility that is not the nearest to their place of residence. Studies from Nepal (1), Tanzania (2, 3), Afghanistan (4), and Uganda (5) have shown that women often circumvent their nearest obstetric care (OC) facility for childbirth. Reports suggest that bypassing by parturients varies in different settings. It can range from 41% in Pwani region, Tanzania (2), to 70% in Nepal (1).

Studies around the world have shown that bypassing a facility for childbirth is associated with maternal and facility characteristics. For example, younger age (1, 3), primiparity (1, 3, 6), obstetric complications (1, 6), and the distance from the mother's home to the nearest facility have been reported as key individual factors increasing the odds of bypassing a facility for childbirth (3, 6). Conversely, frequent antenatal care (ANC) utilization has been reported to be protective against bypassing (1). Mothers also have higher odds of bypassing a facility if their nearest facility was considered to have inadequate human or supporting resources (i.e. infrastructure, medical equipment, or medicines) (7, 8), if it was a private facility (7, 8), and if treatment fees were considered to be high (7, 8). The quality of the care received at obstetric health facilities is also an important determinant of bypassing. Studies in Nepal and Tanzania have shown a positive association between patients’ low perceived quality of care and bypassing (1, 3).

### Why it is important to study the bypassing phenomenon?

Bypassing facilities for childbirth has important implications for service delivery and human resources within a health system. Bypassing dysfunctional lower level facilities can result in overcrowding at the higher level facilities. This can compromise the quality of the services offered at the higher level facilities by overburdening their human and material resources (9, 10). The underutilization of lower level facilities can result in further dysfunctionality because of the loss of skills required to provide emergency obstetric care (EmOC). The dysfunctionality of lower level facilities and the simultaneous overcrowding of those at the higher level impair the ability of the health system to provide timely, efficient, and quality care across different levels of the system.

Over the past decade, various national and subnational programs in India have promoted institutional births, which has resulted in a steep rise in such births (11). The proportion of institutional births in the country has risen from 39% in 2005 (11) to 85% in 2014 (12). The state of Gujarat in Western India has also experienced a similar steep rise in institutional childbirths (13). However, the extent of bypassing in the context of this sharp rise in institutional births has not been studied in India, in spite of its important consequences for the delivery of maternal healthcare services.

We believe that identifying the extent of bypassing, which OC facilities are bypassed for childbirth, as well as assessing the factors influencing such bypassing is important to improve maternal health service delivery and utilization. In this paper, we aim to 1) measure the extent of bypassing health facilities for childbirth by rural mothers in three districts of Gujarat, 2) identify the influence of facility characteristics on bypassing and, 3) assess the relative contribution of maternal and facility characteristics on bypassing.

## Methods

### Study setting

**Fig. 1:**
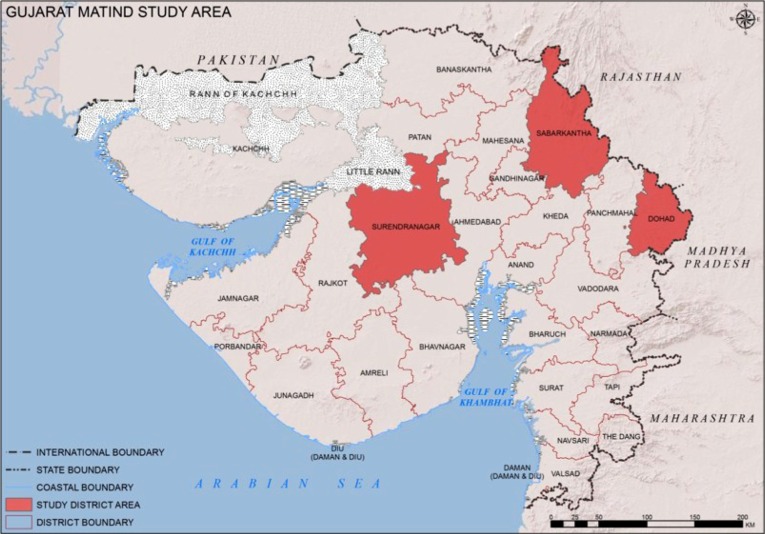
The study area.

The study was conducted in three districts of the state of Gujarat, India (population 60.4 million, 57% rural, and 33% below poverty line) (14). In 2010, the state maternal mortality ratio was 122 per 100,000 live births (15). Gujarat is divided into 26 administrative districts, each with a population of 1–2 million. The three purposively selected districts (Dahod, Sabarkantha, and Surendranagar) included in this study were chosen because they are located in different geographical areas of the state, represent different socio-cultural areas, and have different population subgroups (Fig. 1). Sabarkantha is a relatively wealthier district, whereas Dahod is much poorer and more tribal (Table 1). Surendranagar lies in between.

**Table 1:** Characteristics of the study districts

Characteristics	Dahod	Sabarkantha	Surendranagar
Population (2011)^a^	2,127,086	2,428,589	1,756,268
Proportion rural population (2011)^a^	91%	85%	72%
Proportion literate population^a^	58.8%	75.8%	72.1%
Proportion BPL population^b^	71.6%	32.9%	46.5%

BPL, below poverty level.

a Sample Registrar of India (2011). Districts of Gujarat from www.census2011.co.in/census/state/districtlist/gujarat.html. Socio Economic Survey 2002–03. Add-on lists 2008–09 [database available online].

b Commissionerate of Rural Development, Gujarat. www.ses2002.guj.nic.in/. Accessed 15 January 2015.

OC in the state is provided by both public and private health facilities, with the latter being the dominant provider. Although public facilities provide services that are formally free of charge, the larger private sector operates mainly on the basis of out-of-pocket payments (16). As in the rest of India, pregnant women have access to an accredited social health activist (ASHA). The ASHA is a locally available community-based female volunteer who receives an incentive from the state to facilitate institutional births among pregnant women in her village (17).

### Data collection

#### Facilities

A cross-sectional facility-based study was conducted between June 2012 and April 2013. All OC facilities in the three districts under study were identified from two sources: 1) Gujarat State Health Department and 2) the state professional organization of obstetricians and gynecologists. An initial visit was made to each facility to record the number of births in the 3 months prior to the survey. If a facility reported ≥30 births in this period (166 facilities), then a research team member interviewed the administrator or nurse in charge of the obstetric ward or facility to assess the performance of EmOC signal functions at that facility using a modified version of the Averting Maternal Death and Disability questionnaire (18). In addition, the questionnaire also elicited information on facility characteristics such as ownership, human resources for EmOC, and bed strength.

The functionality of the OC facilities was assessed using the EmOC signal functions as proxy indicators. EmOC signal functions were measured using standard UNFPA definitions (19) (‘key medical interventions that are used to treat direct obstetric complications that cause the vast majority of maternal deaths’). The basic emergency obstetric care signal functions (BEmOC) measured included the following: administration of parenteral antibiotics, uterotonic drugs, parenteral anticonvulsants, manual removal of the placenta, removal of retained products of conception, assisted vaginal delivery, and neonatal resuscitation. Comprehensive EmOC (CEmOC) functions measured were the performance of cesarean sections [CS] and blood transfusions (19). The time frame assessed was the last 3 months. A continuous variable describing the number of signal functions was created to facilitate analysis.

#### Women giving birth

At the same time, a woman research assistant interviewed women giving birth in each facility, with their consent. The interviews were done over five consecutive days in each facility, during which all parturients were invited to participate. Information elicited included age (years), place of residence (urban/rural and, if rural, then village of residence), education (years), possession of a below poverty line card (BPL card, a government-issued formal card that allows the holder to access certain social benefits), number of previous pregnancies (including abortions), number of ANC visits, and caste. Castes in India represent a hierarchical system of social stratification (20). Traditionally, scheduled caste (SC) and scheduled tribe (ST) have been social marginalized and impoverished groups, who benefit from positive affirmative action (21).

For each woman, information on any complications during the current pregnancy (severe anemia, bleeding, multiple pregnancy, fetal malposition, high blood pressure, convulsions, and swelling of body parts) was also obtained from the facility staff. In total, 1,293 women were interviewed, of whom 946 (73.1%) were rural women and thus included in this study.

Geographical information system (GIS) was used to map the position of the mother's residence, the nearest facility, and the facility where she actually gave birth. First, all surveyed facilities and mothers’ villages were georeferenced. Using the georeferenced data, the nearest OC facility from each mother's village was identified. The distance to the actual facility where the mothers delivered was also calculated. If the latter distance was 10 km greater than the distance to the nearest facility (from her village), the woman was classified as a bypasser.

The 10 km cutoff point was chosen because we considered that, in this context, traveling this distance would imply a significantly greater investment of time and resources. OC facilities in Gujarat tend to be clustered in geographical areas, sometimes a few hundred meters distance from each other. Therefore, a mother who chose a facility a few hundred meters away from the nearest one was not considered a bypasser because she did not lose time or travel a significantly longer distance to reach the facility of her choice.

### Data analysis

Data were analyzed using Stata v12 (StataCorp, College Station, TX). Univariate, bivariate, and multilevel statistics were used to analyze the data. Chi-squared and Kruskal–Wallis tests were used to compare bivariate differences between groups. A multilevel mixed-effects logistic regression model was used to account for the clustered nature of our data, and to identify the relative contribution of the characteristics of the facilities to the bypassing phenomenon in Gujarat (22).

As described by Merlo et al. (22), we present the multilevel analysis in three models. Model 1 included only a random intercept and the outcome variable. In this model, the probability of a mother bypassing her nearest facility for childbirth was only a function of the nearest facility itself. This is explained by a facility-level random intercept. The regression function used in model 1 is described as follows: $p1=\frac{\text{Exp}(M+E_{A})}{1+\text{Exp}(M+E_{A})}$ where *M* is the prevalence of the event expressed in the logistic scale and *E*_*A*_ is the facility-level residual on the logistic scale.

In model 2, individual-level variables were added to determine whether the influence of facility differences on the bypassing phenomenon was partly attributable to variations in women's characteristics within each OC facility. Only individual-level variables that were significantly associated with the outcome in the bivariate analysis were included in this model (*p*<0.05). These individual variables included maternal education (years), number of previous pregnancies, number of ANC visits, complications during pregnancy, whether the ASHA accompanied the mother to the health facility and district. The regression function used in model 2 is described as follows: log (pi)=*M*+β_1_education+β_2_pregnancies+β_3_ANC+β_4_complications+β_5_ASHA+β_6_district+*E*_*A*_, where *M* is the prevalence of the event expressed in the logistic scale, *E*_*A*_ is the area-level residual on the logistic scale, and β_1–6_ are the regression coefficients for the maternal covariates.

Model 3 included both individual-level and facility-level variables to assess whether the bypassing phenomenon was associated with specific facility-level characteristics. Individual- and facility-level variables that had a *p* value <0.05 in the bivariate analysis were included. The facility variables added to this model were facility ownership and the number of EmOC signal functions performed in the last 3 months. The regression function used for model 3 is described as follows: log (pi)=*M*+β_1_education+β_2_pregnancies+β_3_ANC+β_4_complications+β_5_ASHA+β_6_district+β_7_facilityownership+β_8_EmOCfunctions+*E*_*A*_, where *M* is the prevalence of the event expressed in the logistic scale, *E*_*A*_ is the area-level residual on the logistic scale, β_1–6_ are the regression coefficients for the individual covariates, and β_7–8_ are the regression coefficients for the facility covariates. Multicollinearity between the variables in the final model was also assessed, and no multicollinearity was found (variable inflation factor was below 10 and tolerance values were above 0.1) (23).

The contribution of the facilities characteristics to the bypassing phenomenon was assessed using two measures of variation: the variance partition coefficient (VPC) and the median odds ratio (MOR). The VPC represents the proportion of the variance (VA) of bypassing that is due to the difference between facilities (22). The MOR is defined as the median value of the odds ratio between the facility at highest risk (of being bypassed) and the facility at lowest risk when randomly picking out two facilities (22). The MOR can be used to quantify facility-level effects on individual health behavior (bypassing) (22). In our study, the MOR shows the extent to which a woman's probability of bypassing her nearest facility is determined by the characteristics of that facility.

### Ethics

Ethical approval was granted by the Indian Institute of Public Health, Gandhinagar, Gujarat, India (reference number: TRCIEC 23/2012) and Karolinska Institutet, Sweden (reference number: 2010/167131/5.I). Informed consent was obtained from all mothers and participating institutions.

## Results

### Facility characteristics

Table 2 describes the characteristics of the facilities. There were fewer privately owned facilities (38.5%). One-third of all facilities had performed a CS or a blood transfusion in the last 3 months. The median number of BEmOC functions performed was 3 (interquartile range [IQR] 2–6). Most facilities were located in Dahod district. Compared to the other districts, a lower proportion of facilities in Dahod district were private. In general, facilities in Dahod district had lower OC functionality than facilities in the other districts (*p*<0.05).

**Table 2:** Facility characteristics stratified by district (*n*=166)

	Sabarkantha (*n*=70)		Dahod (*n*=72)		Surendranagar (*n*=24)		All (*n*=166)	
				
Characteristics	*n*	%	*n*	%	*n*	%	*n*	%
Private ownership – yes^a^	35	50.0	15	20.8	14	58.3	64	38.5
Performed cesarean sections in last 3 months – yes^a^	30	42.8	13	18.0	16	66.6	59	35.5
Performed blood transfusions in last 3 months – yes^a^	26	37.1	11	15.2	12	50.0	49	29.5
Number of basic emergency obstetric care signal functions – median (IQR)^a^	3 (2–5)		2 (1–3)		5 (4–6)		3 (2–6)	

IQR, interquartile range.

a The chi-squared or Kruskal–Wallis tests, *p*<0.05 between the three districts.

### Maternal characteristics

Maternal characteristics are presented in Table 3. Mean maternal age was 24.3 years (standard deviation [SD] 3.6). Mothers had a mean of 5.2 years (SD 4.8) of education, 51.8% belonged to SC or ST castes, and 60.3% had a BPL card. Three in 10 mothers reported no previous pregnancies and 21% had a complication during the current pregnancy. Most mothers (77.3%) had three or more ANC visits, and only 6.5% were accompanied to the health facility by the ASHA (Table 3).

**Table 3:** Women's and facilities’ characteristics stratified by bypassing or not their nearest health facility for childbirth (*n*=946)

		Bypasser	
		
Characteristics	All (*n*=946)	No (*n*=589)	Yes (*n*=357)

Maternal characteristics	No. (%)	No. (%)	No. (%)
District*			
Sabarkantha	417 (44.0)	220 (37.4)	197 (55.1)
Dahod	356 (37.6)	245 (41.6)	111 (31.2)
Surendranagar	173 (18.4)	124 (21.0)	49 (13.7)
Total	946 (100.0)	589 (100.0)	357 (100.0)
Mean age in years (mean/SD)	24.3 (3.6)	24.3 (3.6)	24.3 (3.5)
Years of education (mean/SD)*	5.2 (4.8)	4.7 (4.7)	6.1 (5.0)
Caste			
Scheduled caste	96 (10.2)	61 (10.4)	35 (9.8)
Scheduled tribes	394 (41.6)	256 (43.5)	138 (38.6)
Others	456 (48.2)	272 (46.1)	184 (51.6)
All	946 (100.0)	589 (100.0)	357 (100.0)
BPL card – yes	571 (60.3)	365 (62.0)	206 (57.7)
Previous pregnancies – no*	332 (35.1)	189 (32.1)	143 (40.0)
Complications during pregnancy – yes*	199 (21.0)	106 (18.0)	93 (26.0)
Health service utilization			
Antenatal care visits (three or more)*	731 (77.3)	439 (74.5)	292 (81.3)
ASHA accompanied to health facility*	62 (6.5)	46 (7.8)	16 (4.5)
Distance from the nearest facility (km) (mean/SD)	7.6 (5.1)	7.8 (5.6)	7.7 (5.3)
*Characteristics of nearest facility*			
Ownership*			
Public	594 (62.8)	318 (53.9)	276 (77.4)
Private	352 (37.2)	271 (46.1)	81 (22.6)
Total	946 (100.0)	589 (100.0)	357 (100.0)
Number of EmOC signal functions performed in the last 3 months, median (IQR)*	3 (2–6)	4 (2–7)	2 (2–4)

ASHA, accredited social health activist; BPL, below poverty line; EmOC, emergency obstetric care; IQR, interquartile range; SD, standard deviation.

* The chi-squared or Mann–Whitney tests, *p*<0.05.

### Extent of bypassing and factors associated with bypassing

A little more than one-third of all mothers (37.7%, *n*=357) bypassed their nearest facility for childbirth. Among those who bypassed and whose nearest facility was public (*n*=276), 78.6% (217/276) traveled to a private facility. Nevertheless, of the bypassers whose nearest facility was private (*n*=81), 91.4% (74//81) traveled to another private facility (data not shown). Bivariate analysis showed that bypassing was associated with women's education, having a complication during the current pregnancy, having three or more ANC visits, reporting no previous pregnancies, and not having an ASHA accompany the mother to the health facility (Table 3, *p*<0.05). The proportion of women living in Sabarkantha district was higher among bypassers than among non-bypassers. The proportion of mothers who reported that their nearest facility was public was higher among those bypassing than among those who did not (*p*<0.05). In addition, the median number of EmOC signal functions at the nearest facility was lower among women bypassing than among those who did not (Table 3, *p*<0.05).

### Results from the multilevel models

In building the multilevel model, first (model 1), we included only a facility-level intercept that showed that 50% of the VA of bypassing a facility for childbirth can be explained by the differences between facilities (VPC=0.50, Table 4). It also shows that, in the median case, the residual heterogeneity between facilities increased by 5.78, the maternal odds of bypassing a facility for childbirth when randomly picking two women in different facilities (MOR=5.78, Table 4).

**Table 4:** Multilevel logistic regression of the association between bypassing a facility for childbirth, individual-level and facility-level characteristics; measures of association and clustering are shown (*n*=946)

Measures of association (AOR, 95% CI)	1. Empty model	2. Model with individual-level variables	3. Model with individual-level and facility-level variables
Individual-level variables			
Education (years)	–	1.05 (1.00–1.09)*	1.04 (1.00–1.09)*
Antenatal care visits			
1–2	–	1.00	1.00
3 or more		1.26 (0.79–2.00)	1.32 (0.83–2.09)
Previous pregnancies			
Yes	–	1.00	1.00
No		1.33 (0.92–1.93)	1.33 (0.92–1.92)
Accredited social health activist accompanied to health facility	–		
No		1.00	1.00
Yes		0.51 (0.24–1.11)	0.49 (0.23–1.04)
Complications during pregnancy	–		
No		1.00	1.00
Yes		1.88 (1.22–2.90)	1.86 (1.21–2.88)
District	–		
Sabarkantha		1.00	1.00
Dahod		0.65 (0.30–1.43)	0.32 (0.15–0.66)
Surendranagar		0.37 (0.12–1.09)	0.80 (0.30–2.10)
Nearest facility-level variables			
Ownership	–	–	
Public			1.00
Private			0.62 (0.26–1.44)
Number of EmOC signal functions performed in the last 3 months (unit)	–	–	0.63 (0.53–0.76)
Variance	3.38	3.30	2.01
MOR	5.78	5.66	3.87
VPC	0.50	0.50	0.37

EmOC, emergency obstetric care; MOR, median odds ratio; VPC, variance partition coefficient.

* *p*=0.02.

**Fig. 2:**
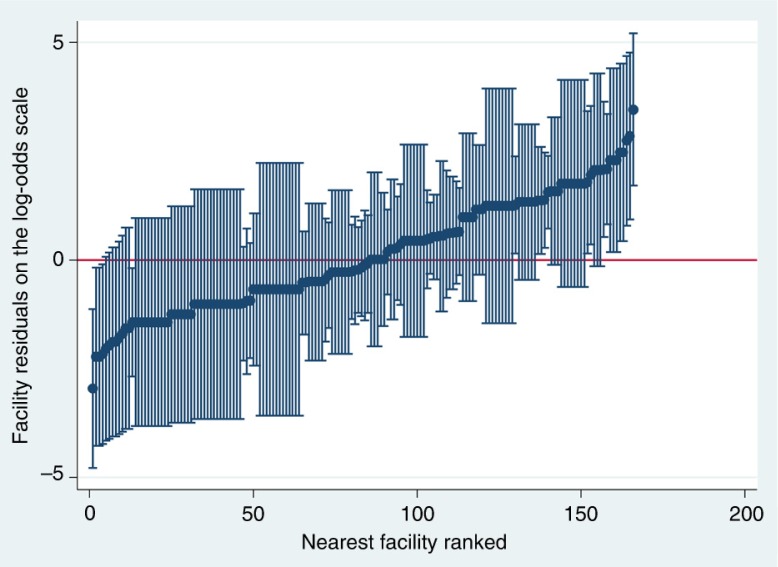
Nearest facility residuals on the log-odds scale ranked from low to high, *n*=166 facilities.

Figure 2 shows the nearest facilities’ residuals and their 95% CI on the log-odds scale ranked from the lowest to the highest odds of being bypassed. The residuals represent facilities departures from the overall residuals mean. Facilities for which the 95% CI of the residuals did not overlap with the mean have either a significantly lower probability (negative log-odds residual values) or a higher probability (positive log-odds residual values) of being bypassed.

Model 2 includes maternal characteristics. It shows that mothers who reported a complication during their current pregnancy had 1.88 higher odds of bypassing their nearest facility for childbirth than those who did not (adjusted odds ratio [AOR] 1.88, 95% confidence interval [CI]: 1.22–2.90). In addition, the model indicated that, for every one-unit increase in the years of education, the odds of bypassing a facility for childbirth increased by 5% (AOR 1.05, 95% CI: 1.00–1.09) (Table 4, model 2).

Model 3 shows that, after adjusting for all individual and facility variables included in the model, maternal factors increasing the odds of bypassing a facility for childbirth were having a complication during childbirth (AOR 1.86, 95% CI: 1.21–2.88), number of years of education (AOR 1.04, 95% CI: 1.00–1.09), and district of residence. The odds of a mother bypassing her nearest facility decreased by 68% if the facility was located in Dahod district instead of Sabarkantha district (AOR=0.32, 95% CI: 0.15–0.66). In addition, for every one-unit increase in the facility's EmOC signal functions, the odds of bypassing a facility for childbirth decreased by 37% (AOR 0.63, 95% CI: 0.53–0.76) (Table 4).

In model 3, the MOR shows that, in the median case, the residual heterogeneity between facilities increased by 3.87 the odds of bypassing the nearest facility when randomly picking two women from different facilities. The residual heterogeneity between facilities (MOR=3.87) was more important than the maternal characteristics to explain the variation in the odds of bypassing a public facility for childbirth. Model 3 VPC shows that 37% of the variation in bypassing the nearest facility can be attributed to the differences between facilities (Table 4).

## Discussion

Our study showed that 4 in 10 rural women bypassed health facilities for childbirth in Gujarat, India. The level of bypassing varied between the districts and was determined by the functionality of facilities, regardless of whether they were public or private. Complications during pregnancy, maternal education, and the district of location were individual factors associated with bypassing. This study showed that variation in a facility's functionality was more important than the maternal characteristics associated with bypassing a facility.

### What facilities are being bypassed?

Studies around the world have reported a link between patient's perceived quality of care and bypassing (1, 3, 24). For example, Karkee et al. elicited women's reasons for bypassing their nearest facility for childbirth (1) by using multiple choice questions. Patients’ perceived quality of care was a strong reason for bypassing, according to that report. However, the use of process indicators is a more objective assessment of the quality of OC (25). Process indicators, such as EmOC signal functions (19), are powerful tools that health systems can and do use to assess health facilities’ preparedness to deal with the main direct causes of maternal morbidity and mortality (25). They also reflect the quality of the obstetric health service, as they can be a proxy measure of the obstetric skills of the staff working in a given health facility.

In this paper, our use of facilities’ performance of the EmOC signal functions as measures of quality allowed us for a more objective measurement. This is relatively new, and has been reported only once before (6). Our multilevel study is the first in South Asia to have found that the number of basic EmOC signal functions performed at a facility was associated with lower odds of it being bypassed. This is a key finding because our study showed that the variation between facilities (MOR=3.87) was more important than maternal characteristics as an explanation of bypassing in this setting. Our results are in line with those recently reported by Kanté et al. in Tanzania (6) and highlight the relevance of using process indicators, such as EmOC signal functions, as predictors of the utilization of obstetric facilities.

Our findings have important implications for health systems. Independent of women's demographic and obstetric characteristics, facilities with low OC functionality had higher odds of being bypassed. Nevertheless, as described by other researchers (9, 10), the underuse of facilities with low obstetric functionality can lead to overcrowding at higher level facilities, with the resulting disruption of OC delivery and decreased quality of care. Thus, it is possible that even though Gujarati mothers with complications bypassed some facilities searching for better OC, the risk of overcrowding facilities with better obstetric functionality might impair the care that they were seeking. However, this study did not evaluate whether these facilities were overcrowded or not; further studies are needed to test this hypothesis.

### Bypassing prevalence and maternal factors

The bypassing prevalence found in our study is similar to the proportions reported from Pwani region, Tanzania (40%) (2), but significantly lower than those reported from Nepal (70%) (1). This difference might be explained by differences in the organization of health systems between the two countries. This could influence the quality, use, availability, distribution, and the type of OC facilities. For example, 9 in 10 childbirths are institutional in Gujarat compared to only 4 in 10 institutional births in Nepal (26). In Nepal, women who have institutional childbirths are wealthier than those who do not (26), suggesting that the few women giving birth in health facilities were more able to choose where to give birth. This might explain the high bypassing rate reported by Karkee et al. (1).

Our study also found that geographical location was another important determinant of bypassing. The lower odds of bypassing found in Dahod district suggest that bypassing can also be determined by differences in the overall availability of comprehensive OC between districts. As our previous study has shown (27), comprehensive OC facilities and facilities offering CS, but not all other signal functions, are concentrated mainly in Sabarkantha district, with very few in Dahod. In addition, as our results indicated, most OC facilities in Dahod have low OC functionality, which means that they offer fewer EmOC signal functions. Thus, compared to mothers living in Sabarkantha district, mothers living in Dahod district have less access to comprehensive OC. They face a homogeneous group of facilities with similar low levels of obstetric care. Thus, rather than being a positive finding, the lower odds of bypassing found for Dahod district are more likely to reflect the mothers’ relatively poor access to comprehensive OC, compared to mothers living in Sabarkantha.

Our results indicated that having a complication during the current pregnancy increased the odds of bypassing a facility for childbirth. This is in line with studies conducted in other low- and middle-income countries that report a direct association between illness severity (8), obstetric complication during the current (1) or a previous childbirth (6), and the bypassing of facilities for health care. The higher odds of bypassing might reflect mothers’ and their families’ increased awareness of the need for specialized care that might not be available at their nearest point of OC. Higher level of maternal education was also associated with bypassing in this setting, which is consistent with the findings from a study conducted in Afghanistan (4). Mothers with higher educational levels might have a better socioeconomic status, which allowed them to cover the extra expense associated with bypassing their nearest facility for childbirth.

### Limitations

This study has an important limitation. Given the heterogeneity of OC provision in other districts of Gujarat, our study findings cannot be generalized to other districts in the state or the country.

## Conclusions

Our study concluded that 4 in 10 women bypassed health facilities for childbirth in Gujarat, India. The level of bypassing varied between the districts and was determined by the functionality of facilities, whether public or private. This study showed that variations in facilities’ functionality were more important factors associated with bypassing a facility for childbirth than maternal characteristics. It is clear that, for bypassing rates to decrease in Gujarat, efforts must be made to improve the obstetric functionality of facilities that are nearest to the mothers’ villages. In addition, inequalities in EmOC availability and quality between districts must be addressed.

## Paper context

Bypassing health facilities for childbirth is a common phenomenon that can disrupt effective obstetric service delivery. India has experienced a steep rise in facility births; however, the magnitude and determinants of bypassing in this setting are unknown. This study found that bypassing was common and was associated with the obstetric functionality of facilities. For bypassing rates to decrease in Gujarat, efforts must be made to improve the functionality of the facilities that are nearest to the mothers’ villages.
